# Evaluation of self-administered tests for pelvic girdle pain in pregnancy

**DOI:** 10.1186/1471-2474-15-138

**Published:** 2014-04-27

**Authors:** Monika Fagevik Olsén, Helen Elden, Annelie Gutke

**Affiliations:** 1 Department of Physical Therapy and Occupational Therapy, Sahlgrenska University Hospital, Gothenburg SE 413 45, Sweden; 2 Department of Physical Therapy and Occupational Therapy, Institute of Neuroscience and Physiology Sahlgrenska Academy, University of Gothenburg, Gothenburg, Sweden; 3 Institute of Health and Care Sciences, Sahlgrenska Academy, University of Gothenburg, Gothenburg, Sweden

**Keywords:** Agreement, Pelvic girdle pain, Pregnancy, Tests

## Abstract

**Background:**

Different tests are used in order to classify women with pelvic girdle pain (PGP). One limitation of the tests is that they need to be performed by an examiner. Self-administered tests have previously been described and evaluated by women who performed the tests directly before the examiner performed the original tests. Thus, an evaluation of the self-administered tests performed in a more natural setting, such as the women’s home is needed.

The purpose of this study was to investigate the agreement between self-administered tests performed at home and tests performed by an examiner on women with suspected PGP. Additionally to compare the classification made by an examiner and classification based on results of the self-administered tests and questionnaire.

**Methods:**

One hundred and twenty three pregnant women with suspected PGP participated. Before the appointment at the clinic the women performed the self-administered tests and filled in a questionnaire. During the appointment one specialized physiotherapist performed the tests. Result of the two different sets of tests and the classifications made by the examiner and the self-administered tests including questionnaires were compared concerning percentage of agreement (POA), sensitivity and positive predicted value (PPV).

**Results:**

The P4 and the bridging test had the highest POA (≥74.8%), sensitivity (≥75.5%) and PPV (≥91.2%) for posterior PGP. For anterior PGP the MAT test had highest POA (76.4%), and PPV (69.5%), and the modified Trendelenburg test the highest sensitivity (93.0%). Agreement between the two classifications was 87%.

A significantly higher number of positive P4 and bridging tests (p < 0.01) and a significantly lower number of positive Trendelenburg tests, Active Straight Leg raise and Straight Leg Raise (p < 0.05) were recorded by the examiner compared to the self-administered ones.

**Conclusions:**

Our results indicate that self-administered test and questionnaires are possible to use for testing and classification of women with suspected PGP.

## Background

Lumbopelvic pain is one of the most common complications of pregnancy [1]. The most frequent pain location and the most severe pain are related to the pelvic girdle [2]. Posterior pelvic girdle pain (PGP) has been defined as pain localized between the iliac crests and the gluteal folds with or without radiation down the leg [3]. Anterior PGP is experienced in the symphysis and can occur in addition to posterior PGP or as a separate syndrome, often termed symphysiolysis.

PGP is provoked or increased by everyday activities such as walking, standing, sitting and lying down [4,5]. It has been shown that PGP can increase after as little as 30 minutes of activity, which limits most daily activities and the ability to work [5]. At the individual level, consequences such as decreased health-related quality of life and a higher proportion of depressive symptoms are seen [6,7]. At the societal level, consequences are seen in high sick leave costs, with lumbopelvic pain standing for the main part of the social benefits for pregnant women [1].

Since effective treatments have been described [8,9], it is important to identify women who suffer from PGP. According to guidelines, provocation tests are needed to identify PGP and to exclude lumbar causes, but there is no consensus concerning which tests to choose [3]. Identification of women with severe PGP is also important since they have the highest risk of persistent pain both during [10] and after [11] pregnancy. Among the large group of women with lumbopelvic pain in pregnancy, women with PGP have reported the greatest consequences in terms of pain intensity, disability and health-related quality of life [2]. Outcomes of clinical tests has been shown to predict risk of persistent pain [11,12], emphasizing the importance of examination in addition to solely a pain drawing and questions about pain bearing activities when used for screening of PGP in trials and in clinical practice.

Large surveys must be done to learn more about the aetiology and incidence of PGP, and to identify the relatively few women with severe persistent PGP [13]. Likewise, when doing longitudinal studies or follow-up studies after treatment, it could be an advantage to have a practical and inexpensive way to screen for PGP. Large surveys are expensive and diagnosis is usually defined by pain drawings and questionnaires. Since there is uncertainty as to whether women with PGP can be identified by questionnaires alone, an initial screening for PGP using self-administered tests may be suitable. These tests may increase the chance of more specifically identifying women with PGP. Self-administered tests could also be used in perinatal care where midwifes can ask women with suspected PGP to perform the tests under supervision. The information from the results of the tests can guide midwifes when they advice these women and refer them to physical therapy and other treatments.

To investigate the possibility to use a self-administered test, tests were developed based on frequently used clinical tests recommended by the European Guidelines [3,14]. As several tests are recommended for a more reliable diagnosis [15], a series of tests was developed. An initial study was done with the aim to examine which self-administered tests that were most sensitive and specific and had the highest percentage of agreement in pregnant women with and without PGP [14]. In that trial, the women performed the tests after verbal instructions, at the clinic directly before the standardised tests were performed by an examiner. The results indicated that pregnant women can perform a screening by provocation the pain by self-administered tests. However, an evaluation of the agreement of the tests performed after written instructions in a more natural setting than the clinic, such as home is valuable.

The purpose of this study was to investigate the agreement between self-administered tests performed at home and tests performed by an examiner in a clinic on women with suspected PGP. Additionally we wanted to compare the classification made by an examiner and the classification by self-administered tests combined with response to questionnaire.

## Methods

A consecutive series of 160 pregnant women referred from antenatal centres to a specialist clinic for suspected PGP were asked to fill in questionnaires before the visit at the clinic. The questionnaire included questions about the background, intensity and duration of PGP and a pain drawing. The women also received a form with information about how to perform the self-administered tests including instructive photos. The women were asked to perform the tests the evening before their appointment at the clinic. Of the 160 women 123 (77%) performed all tests and filled in the questionnaires before the visit.

**Figure 1:**
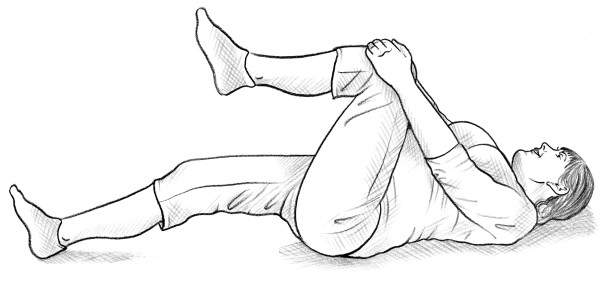
Self-administered P4 test.

**Figure 2:**
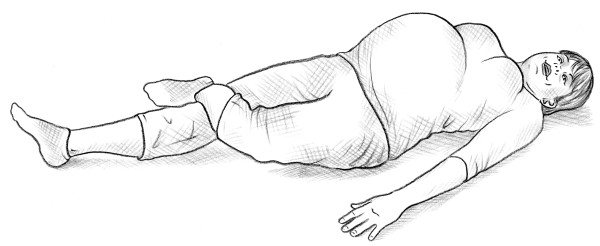
Self-administered Patrick Faber test.

**Figure 3:**
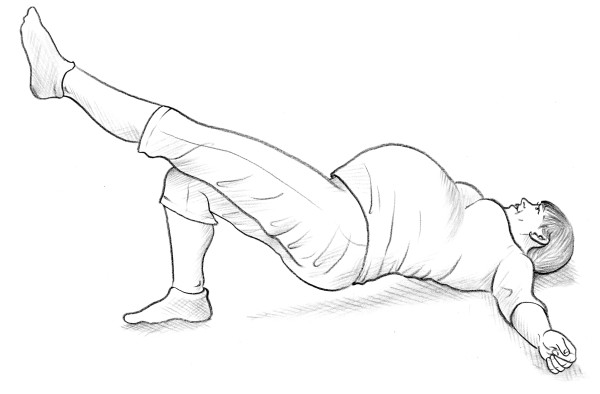
Bridging test.

**Figure 4:**
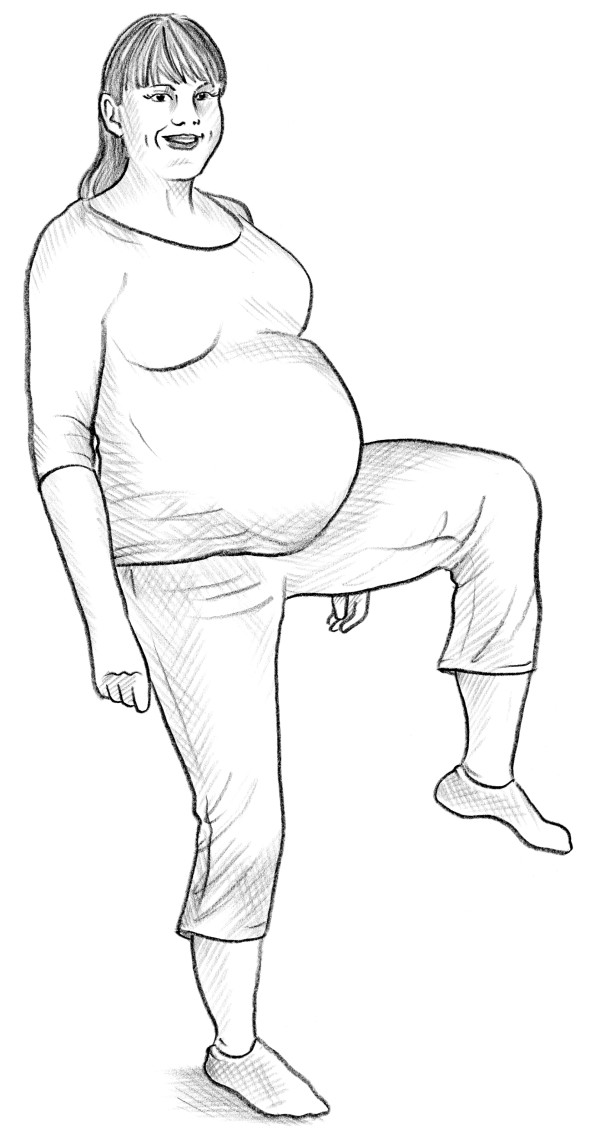
Modified trendelenburg test.

**Figure 5:**
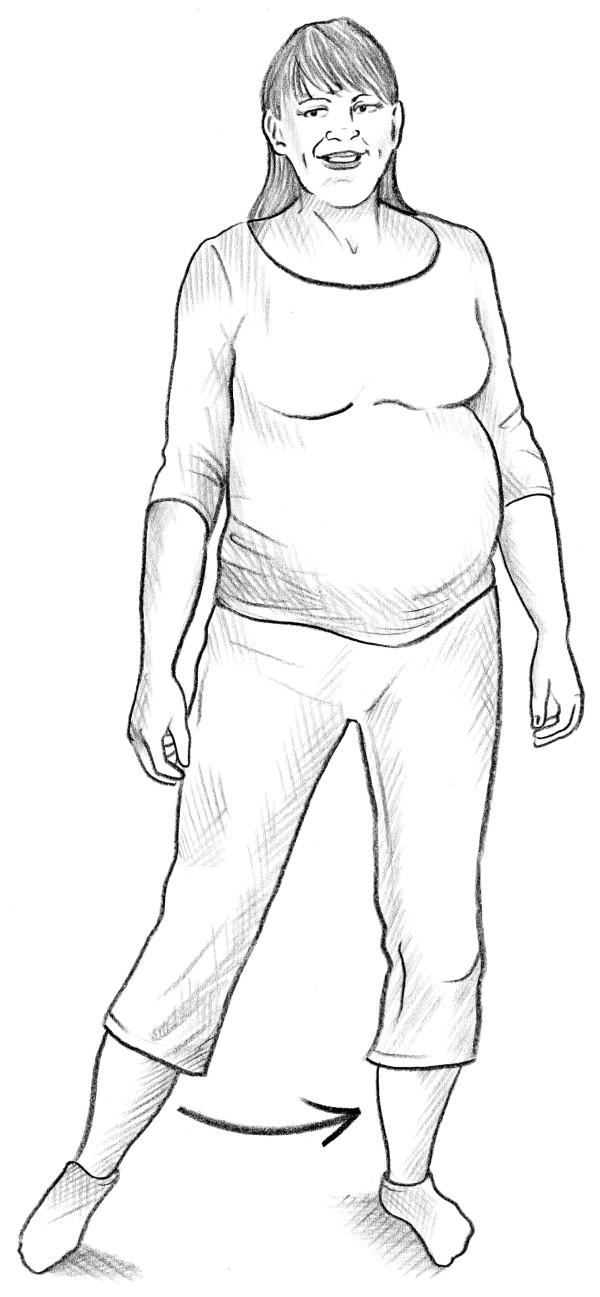
MAT test.

The following self-administered tests were performed on the floor by all of the women, once for each leg, and the absence or presence of familiar pain was noted:

Pain provocation tests:

• The self-administered posterior pelvic pain provocation test (P4 test) [14] (Figure 1).

• The self-administered Patrick Faber test [12] (Figure 2).

• Bridging test [14] (Figure 3).

• The self-administered Trendelenburg test [12] (Figure 4).

• MAT test [14] (Figure 5).

**Figure 6:**
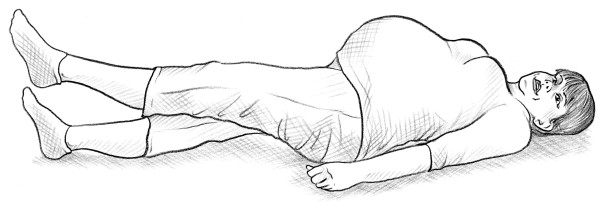
Self-administered ASLR test.

Functional test:

• The self-administered active straight leg raise test (ASLR) [16] (Figure 6).

**Figure 7:**
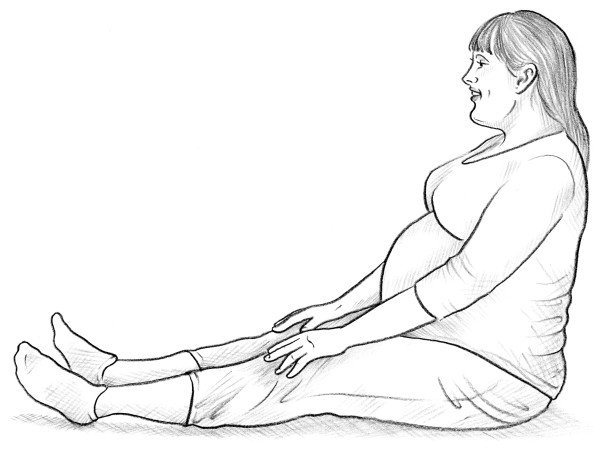
Self-administered modified SLR test.

In addition, to be able to evaluate possible nerve affection a self-administered straight leg raise test was performed (Figure 7).

During the clinical visit, one examiner did a standardised examination of all the women, including pain provocations of the back and pelvis. The instructions to the women were the same as in the written instructions for the self-administered tests. The presence/absence of pain was recorded. The examiner did not know the results of the self-administered tests when performing the examination at the clinic.

**Figure 8:**
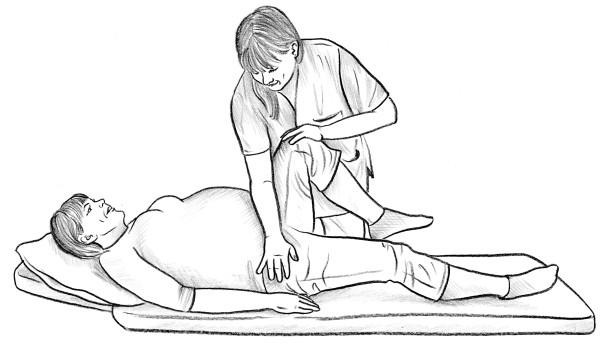
P4 test.

**Figure 9:**
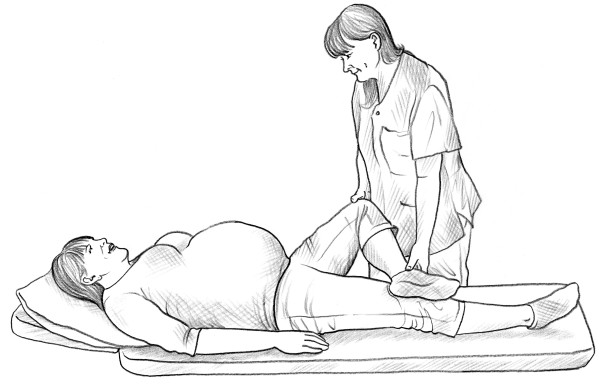
Patrick Faber test.

Pain provocation tests:

• The posterior pelvic pain provocation test (P4 test) [17] (Figure 8).

• Patrick Faber test [12] (Figure 9).

• Bridging test [14] (Figure 3).

• The modified Trendelenburg test [12] (Figure 4).

• MAT test [14] (Figure 5).

• Palpation of the symphysis [12].

**Figure 10:**
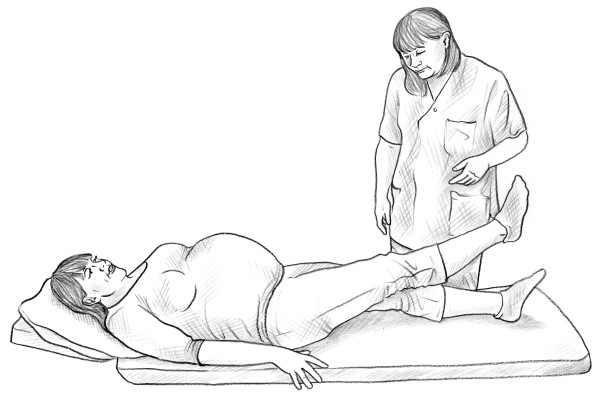
ALSR test.

Functional test:

• ASLR, the women rated difficulty in raising one leg on a scale from 0–5 [16] (Figure 10).

**Figure 11:**
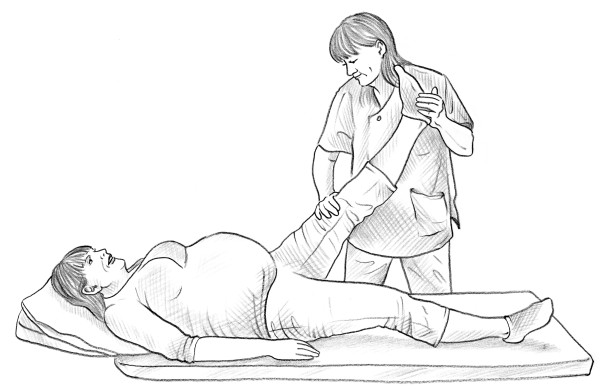
SLR test.

Nerve tension test:

• Straight leg raise [18] (Figure 11).

All tests were performed once for each leg. To verify the pain/absence of pain, the women were interviewed before the examination about daily symptoms in their pelvic girdle and lower back.

The classification of PGP during the clinical visit was made according to the definition in the European guidelines [3]. All criteria had to be fulfilled.

• Pain experienced between the posterior iliac crest and the gluteal fold, particularly in the vicinity of the sacroiliac joints in conjunction with/or separately in the symphysis.

• Reports by the women of weight-bearing related pain and its duration in the pelvic girdle.

• Diminished capacity to stand, walk and sit.

• Positive clinical diagnostic tests, which reproduced pain in the pelvic girdle.

• No nerve root syndrome (Negative SLR test).

Classification of PGP based on the results of the self-administered tests and questionnaires was as followed:

• A pain drawing with well defined markings of pain over the gluteal area or the symphyseal joint.

• A history of weight-bearing related pain in the pelvic girdle.

• Positive self-administered tests, which reproduced pain in the pelvic girdle.

• No nerve root syndrome judged by a negative self-administered modified straight leg raise.

### Statistics

The proportion of positive and negative tests during the test at home and at the clinic was analysed by McNemar’s test. The two versions of each test were analyzed for:

• Percentage of agreement (number of patients where the two versions of the tests were in accordance/number of all tested women).

• Sensitivity (number of patients where both versions of the tests were postive/number of women with positive test at the clinic).

• Positive predictive value (PPV) (number of patients where both versions of the tests were postive/number of women with positive self-administered test).

For calculation purposes, the tests performed at the clinic were used as the reference standard to which the self-administered tests were compared.

The ASLR was analysed with the Wilcoxon Signed rank test for scores from 0–10 and McNemar’s test, where the scores were dichotomised to “positive” for scores between 1 and 10 or “negative” for score 0.

While it is not possible to perform palpation of the symphysis as a self-administered test because of the difficulty in standardising the pressure, the percentage of agreement between palpation during the visit and the self-administered MAT test was also analysed. According to the results of our previous trial [14], the self-administered P4 test had lower percentage of agreement than the bridging test in comparison with the P4 performed by an examiner. The sensitivity of the self-administered bridging test and the P4 test performed by the examiner was therefore also analysed. In addition, the percentage of agreement, sensitivity and PPV for the classification set during the visit and the one based on the women’s self-administered tests and questionnaires were analysed.

The regional Ethic Committee in Gothenburg approved the study protocol (Registration number: 099–09). The patients were included after oral and written information and written consent.

## Results

The 123 women who performed the self-administered tests before the visit to the clinic were on average 30.7 (SD 4.5) years of age, in gestational week 22 (SD 4.7) and pregnant with their second child (min 0- max 4). The women were well distributed as concerns educational level and sedentary vs. active lifestyle.

Results of the self-administered tests and the tests performed at the clinic are given in Table 1. There were significantly higher numbers of positive P4 and bridging tests during the visit compared to positive self-administered tests (P = 0.036 and 0.001 respectively). There were significantly lower numbers of positive modified Trendelenburg tests (anterior p < 0.001, posterior p < 0.016) ASLR and SLR (both p < 0.001) during the visit compared to positive self-administered tests.

**Table 1:** Number of positive self-administered tests performed at home and positive tests performed by an examiner on women with suspected PGP and classification made by an examiner and based on results of the self-administered tests plus questionnaire

**Test**	**Positive test during the visit,**	**Positive self-administered test,**	**P-value between the groups**
	**n = 123**	**n = 123**	
**Posterior pain**			
Positive P4, n	103	91	0.036
Positive Patrick Faber test, n	73	83	0.174
Positive, modified Trendelenburg test, n	54	71	0.016
Positive bridging test, n	102	83	0.001
**Anterior pain**			
Positive palpation of the symphysis, n	60	n.a	
Positive modified Trendelenburg test, n	43	97	<0.001
Positive MAT test, n	52	59	0.265
**Additional tests**			
ASLR, 0-10	2 (0–10)	4 (1–10)	<0.001
Positive ASLR (≥1), n	83	105	<0.001
SLR, n	0	23	<0.001
**Diagnosis**			
Fulfilling the criteria for classification of posterior pain, n*	103	99	0.481
Fulfilling the criteria for classification of anterior pain, n*	52	50	0.845

N or median (min-max).

n.a. not applicable.

*Classification during the clinical visit: pain experienced between the posterior iliac crest and the gluteal fold or in the symphysis, weight-bearing related pain, diminished capacity to stand, walk and sit, positive clinical diagnostic tests and no nerve root syndrome [3]. Classification based on the results of the self-administered tests and questionnaires: pain drawing with well defined markings of pain over the gluteal area or the symphyseal joint, a history of weight-bearing related pain in the pelvic girdle, positive self-administered tests and no nerve root syndrome.

The percentage of agreement, sensitivity and PPV between the self-administered tests and the tests done by the examiner during the visit was calculated. Results are given in Table 2. Of the evaluated tests for posterior PGP the P4 and bridging tests had the highest percentage of agreement (77.2 and 74.8%), sensitivity (80.6 and 75.5) and PPV (91.2 and 92.8%). Of the two tests for anterior pelvic pain the MAT test had the highest percentage of agreement (76.4%) and PPV (69.5%) but the modified Trendelenburg test had the highest sensitivity (93.0%).

**Table 2:** The proportion of positive and negative tests, percentage of agreement (POA), sensitivity and PPV

**Test**	**Both +**	**Both -**	**Visit + home -**	**Visit - home +**	**POA**	**Sensitivity**	**PPV**
**Posterior pain**							
P4	67%	10%	16%	7%	77.2	80.6	91.2
Patrick Faber test	46%	19%	14%	22%	64.2	76.7	67.5
Modified Trendelenburg test	33%	31%	11%	25%	51.3	56.3	74.1
Bridging test	63%	12%	20%	5%	74.8	75.5	92.8
**Anterior pain**							
Modified Trendelenburg test	33%	18%	2%	47%	50.4	93.0	40.8
MAT test	33%	43%	9%	15%	76.4	78.8	69.5
**Additional tests**							
ASLR, 0-10					38.2	78.9	
Positive ASLR (≥1), n	66%	13%	2%	20%	78.9	97.6	77.1
SLR	0%	81%	0%	19%	81.3	0	0
**Diagnosis**							
Fulfilling the criteria for diagnosis	83%	4%	7%	6%	87.0	91.9	93.6

+ positive test, − negative test, both = results from test at home and during the visit.

The percentage of agreement between P4 performed by an examiner and the self-administered bridging test was 78% and the sensitivity 77%. The percentage of agreement and sensitivity between the palpation of the symphysis and the self-administered MAT test were found to be 65% and 67%.

Of the 123 women with a positive pain drawing and pain history according to the questionnaire, 109 also had positive self-administered tests. One hundred and eleven women were classified with PGP by the examiner. There was no significant difference between the proportion of women who were classified with PGP by the self-administered tests and questionnaire and during the visit (p = 0.845). Of the 118 women classified with PGP either based on results from self-administered tests and response to questionnaire or based on classification of the examiner, nine were classified only by self-administered tests combined with response to questionnaire, seven only during the visit. The agreement between both classifications of the examiner and what was reported in the self-administered tests combined with questionnaires was 87% (n = 102) (Table 2).

## Discussion

The main findings of this study are that self-administered test and questionnaires are possible to use for testing and classification of women with suspected PGP. In our earlier trial [14], where both pregnant women with and without pain and non-pregnant women without pain were assessed the results indicated that the self-administered tests had high sensitivity and specificity. Based on both our trials, the tests and concept seems to be usable in larger surveys. In addition, they can be used in perinatal care units as a ground for referral to physical therapy or other treatments for pregnant women with suspected PGP. This could also reduce the mistrust which may occur between midwives and pregnant women if vague symptoms are reported [19].

Among the tests for identification of posterior PGP, the highest percentage of agreement and sensitivity was seen for the self-administered P4 test as compared to the traditional P4. The result is in accordance with previous studies [12,20]. A reason for the high agreement may be the standardisation of the test and simplicity of its performance. Likewise, this may explain why the MAT test showed the highest percentage of agreement among the tests for identifying anterior PGP.

The bridging test is another assessment for identifying posterior PGP that has been shown to have a high sensitivity and high percentage of agreement compared to tests performed by an examiner. In our previous study, the bridging test had a higher sensitivity than the self-administered P4 test when compared to the traditional P4 test [14]. In the current study, the sensitivity of the self-administered and examiner performed P4 test was 80.6% and the bridging test 75.5%. This indicates that it may be an advantage to use at least these two self-administered tests for identification of PGP, as it has been reported that two to three positive pain provocation tests are required for a clinical classification [15]. On the other hand it is important to limit the number of tests used while the test may trigger the pain. It seems like it is enough to use the P4 and bridging test to encompass the posterior pain.

The ASLR test was included in this evaluation because it is used as a functional test to determine the load transfer between legs and lumbar spine. [16]. The results indicate though that it is less suitable as a self-administered test, as some women gave a score for difficulty in lifting the leg at home and less difficulty when the test was repeated at the clinic. A possible explanation for our findings could be the test’s grading system, where total concurrence is harder to fulfil. A further analysis was then performed where the results of the tests were dichotomized. The percentage of the agreement was then 78.9% between the tests indicating that if the test is used self-administered it is better to ask if the patient has difficulties to raise the leg or not than to grade the difficulty from 0–5. None of the women had a positive SLR during the visit, but 23 registered a positive self-administered SLR test. A possible explanation may be that the self-administered SLR test gave unspecific muscle pain, which the women interpreted as radiating pain to the foot. A positive nerve root pain is rare among pregnant women and, to avoid false positive self-administered tests, a better description is needed of how to interpret pain in the test.

The women in our study were included at a specialist clinic for lumbopelvic pain and our results might be generalised to women who seek care for their pain during pregnancy. Among these women, there are probably many with a high risk of persistent pain postpartum [21,22], since women with severe pain and disability are more likely to seek care for their symptoms than women with mild complaints. Our results are promising for women who need to be identified early for treatment. Patients with verified pain can then be referred for further examination and treatment.

The tests evaluated in this article were chosen according to recommendations in guidelines and clinical trials concerning tests [3,12,17]. However, there are several other tests for PGP that were not included. It may be possible to use some of them in a self-administered way by the women, with or without adjustments. In addition, there is a need for self-administered tests for other structures close to the pelvis that can cause pain during pregnancy, such as the hip joint and groin.

There were significantly higher numbers of positive P4 and bridging tests during the visit compared to the self-administered tests (P = 0.036 and 0.001 respectively) and significantly lower numbers of positive modified Trendelenburg tests during the visit (anterior p < 0.001, posterior p < 0.016). The larger number of positive tests at the clinic may be explained by a more specific test procedure during the P4 and bridging tests. The interaction between examiner and subject can also be a reason while there is a risk that the patients try to communicate to the examiner that there definitely is a problem, making tests more positive. The explanation for the discrepancy concerning the modified Trendelenburg test may be that unspecific pain in the pelvic region can be misinterpreted by women to be symphyseal or PGP.

There are two other reasons that may explain the discrepancy between the tests. In this study, the self-administered tests and tests performed during the standardised examination were not performed on the same day. In our earlier study [14], the women performed both sets of tests at the clinic. PGP is reported to be more severe during evenings [9,23] and the discrepancy between the two series of tests may be caused by that the women performed the self-administered test in the evening and the test at the clinic was performed in mornings or afternoons. However, as the repeated tests may overload the structures and trigger PGP, thus giving false positive results, it can be an advantage not to repeat the tests on the same day. Another explanation may be that the self-administered tests were performed according to written instructions and photos. In our earlier trial, verbal instructions were given and the women could ask for further instructions when they needed them. In an attempt to standardise the tests in the current evaluation, the women were instructed to perform the tests on the floor so that they were on a solid surface, rather than doing the tests in a soft bed. The same instructions were given at the clinic.

One limitation of this study is that women with pain of discogenic origin may not have been identified but such origin of lumbopelvic pain in pregnancy is rare [24]. Another limitation is that this trial was undertaken to evaluate the tests in women who was referred to a specialist clinic because of suspected PGP and not a cohort of pregnant women. However, it is a first evaluation of the tests performed by women themselves in a natural setting, eg their homes. More evaluations are needed to explore the tests usability in pregnant women with and without lumbopelvic pain and functional limitations. The tests usefulness for classification of PGP postpartum also needs to be evaluated further.

Since it seems possible to identify women at risk for persistent PGP after pregnancy already early in the pregnancy [25,26], the self-administered test could contribute to specific identification of PGP and thereby provide the basis for an early intervention.

## Conclusions

Our results indicate that self-administered test and questionnaires are possible to use for testing and classification of women with suspected PGP.

## Competing interests

The authors declare that they have no competing interests.

## Authors’ contributions

All authors have made equal contributions to conception and design, analysis and interpretation of data. All authors have also been involved in drafting the manuscript and given final approval of the version to be published.

## Pre-publication history

The pre-publication history for this paper can be accessed here:

http://www.biomedcentral.com/1471-2474/15/138/prepub
